# Radiation-driven rotational motion of nanoparticles

**DOI:** 10.1107/S1600577518005039

**Published:** 2018-04-25

**Authors:** Mengning Liang, Ross Harder, Ian Robinson

**Affiliations:** a Linac Coherent Light Source, SLAC National Accelerator Laboratory, 2575 Sand Hill Road, MS103, Menlo Park, CA 94025, USA; b Advanced Photon Source, Argonne National Laboratory, Argonne, IL 60439, USA; c Centre for Nanotechnology, University College, London, London WC1H 0AH, UK; d Condensed Matter Physics and Materials Science Department, Brookhaven National Laboratory, Upton, NY 11973, USA

**Keywords:** rotational X-ray tracking, radiation pressure, rotational dynamics

## Abstract

Focused synchrotron beams can influence a studied sample *via* heating, or radiation pressure effects due to intensity gradients. The high angular sensitivity of rotational X-ray tracking of crystalline particles *via* their Bragg reflections can detect extremely small forces such as those caused by field gradients. By tracking the rotational motion of single-crystal nanoparticles embedded in a viscous or viscoelastic medium, the effects of heating in a uniform gradient beam and radiation pressure in a Gaussian profile beam were observed. Changes in viscosity due to X-ray heating were measured for 42 µm crystals in glycerol, and angular velocities of 10^−6^ rad s^−1^ due to torques of 10^−24^ N m were measured for 340 nm crystals in a colloidal gel matrix. These results show the ability to quantify small forces using rotation motion of tracer particles.

## Abstract 2

The observation of the effects of radiation pressure of a Gaussian beam by tracking the rotation motion of single-crystal nanoparticles is presented.

## 1. Introduction   

Measurement of the rotational motion of nanometer-sized tracer particles is a useful way to measure viscous and viscoelastic media (Cheng & Mason, 2003 ▸; Andablo-Reyes *et al.*, 2005 ▸). Consider a small single crystal suspended in a fluid exposed to an X-ray beam of intense synchrotron radiation. When it happens to be oriented at its Bragg angle, there will be measureable diffraction intensity on an area detector aligned at the proper 2θ scattering angle. Should the crystal rotate about an axis parallel to the incident beam, the Bragg condition is maintained, and the resulting change in position of the diffraction peak can be monitored with extremely high sensitivity. Interpreting the resulting motion of the diffraction signal on the detector as a function of time is the mechanism behind the technique of Rotational X-ray Tracking (RXT) (Liang *et al.*, 2014 ▸). The rotational mean-squared displacement (MSD) of the particle can reveal important microscopic details about the system – a purely viscous medium would have a linear MSD with a slope based on the viscosity, whereas the presence of a driving force on the particle would introduce a quadratic dependence, and a complex functional dependence would indicate a viscoelastic medium.

RXT can have a resolution of tens of microradians, three orders of magnitude higher than that of optical particle tracking techniques (Anker & Kopelman, 2003 ▸; Fakhri *et al.*, 2010 ▸). The time and angular resolution of RXT is dependent on both the pixel size and readout rate of the detector and the intensity of the incident X-ray beam. There must be sufficient photons scattered and detected within the time span of interest to identify the position of the Bragg peak. This suggests that higher flux is required for better time resolution. Higher flux, however, means increased radiation effects in terms of heating, radiation pressure and damage. In order to explore these effects in more detail, we performed systematic studies to understand how beam parameters effect the rotational motion of particles in suspension.

The rotational motion of particles can be affected by an X-ray beam in three major ways: (1) heating of the entire system by the beam, which causes a local change in the thermal energy of the system, and can also change the viscoelastic properties of the surrounding medium; (2) radiation pressure due to a non-planar X-ray wavefront which induces a torque on particle; or (3) damage to the system, medium or particle, which is typically irreversible. The topic of radiation damage has been an area of much interest and effort, experimentally and theoretically, especially in the context of protein crystallography, so we refer to the established literature (Warkentin *et al.*, 2011 ▸; Holton, 2009 ▸).

In the context of Brownian motion, rotational motion of a probe particle has a third-power size dependence as compared with a linear dependence for translational motion (Perrin, 1928 ▸). Such dependence means that, for a small particle, the rotational motion is especially sensitive to any changes in viscosity due to heating, or to small torques due to radiation pressure. Less than an attonewton of radiation pressure force has been observed on gold nanocrystals attached to substrates with actin filaments using diffractive X-ray tracking (Sasaki *et al.*, 2000 ▸) and radiation pressure was observed to act on Pd nanocubes and a Ni nanowire (Kim *et al.*, 2016 ▸). The rotational motion seen by RXT at a third-generation source with a standard detector is sensitive to torques of order 10^–24^ N m, as computed in a later section of this article, providing the necessary sensitivity to observe radiation pressure force gradients. Here we study, and quantify, the effects of synchrotron X-rays on the rotational motion of particles with respect to heating and radiation pressure by studying the motion at different X-ray fluxes for both temperature sensitive and temperature independent systems to decouple the effects of temperature and radiation pressure.

## 2. Radiation heating   

**Figure 1:**
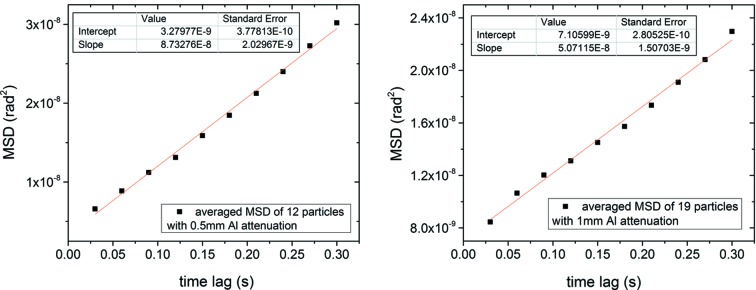
MSD *versus* time for 42 µm alumina particles in glycerol at two different fluxes. The different slopes reflect the difference in viscosity due to X-ray heating.

To investigate radiation heating, we chose glycerol, which has a strong temperature-dependent viscosity, and performed X-ray flux studies of the rotational motion of 42 ± 3.5 µm alumina crystals in a planar X-ray beam profile. The sample was measured in a sealed capillary in transmission geometry at the X11 beamline at Doris III with an unfocused 15.2 keV beam of 10^13^ photons s^−1^ in a spot size of 1 mm × 1 mm. Attenuators were employed to reduce the incident photons in a controlled way. Diffraction of the (104) Bragg reflection of alumina was measured with a Mythen strip detector with time steps of 30 ms and 50 ms at a sample to detector distance of 0.7 m. The angular MSD *versus* time 〈θ^2^(*t*)〉 was measured for different X-ray flux levels using RXT data analysis methods as shown in Fig. 1 ▸.

The differences in the viscosities are calculated from the MSD, assuming rotational diffusion for spherical particles 〈θ^2^(*t*)〉 = *k*_B_ *T*/8πη*R* ^3^, where η is the dynamic viscosity and *R* is the radius. The resulting values are 7.9 × 10^−1^ Pa s for the case of 1 mm aluminium (Al) attenuation in the X-ray beam (14% transmission) and 4.2 × 10^−1^ Pa s for the 0.5 mm Al attenuation of the X-ray beam (35% transmission). For the 99% pure glycerol used, the viscosity value measured with the greater X-ray attenuation (1 mm Al) corresponds to a temperature of 27°C and the more intense beam (lower attenuation) corresponds to 33.5°C, based on known data for the change in viscosity as a function of temperature and the difference in thermal energy (*k*_B_ *T*).

Such temperature increases are consistent with previous studies on heating with synchrotron beams (Snell *et al.*, 2007 ▸). Heating can also lead to convection or flow effects, which cause deviations from linear behavior in the MSD *versus* time graph by the addition of a quadratic component. At a time lag of 0.3 s, some deviation is observed; however, the largely linear trend of MSD *versus* time at times shorter than 0.3 s allows us to conclude that the motion at these shorter time lags is primarily diffusive.

## 3. Radiation pressure   

Radiation pressure was observed over a century ago in optical light (Nichols & Hull, 1903 ▸; Frisch, 1933 ▸) and has been utilized in laser cooling for atomic traps (Phillips, 1998 ▸), but the relatively low power of X-ray beams compared with optical lasers has limited the study of X-ray radiation pressure mainly to astrophysics (Nichols & Hull, 1903 ▸). Radiation pressure due to a planar beam would result in a force that would drive a particle in translation but should not cause rotation. However, a beam with a non-uniform intensity profile could induce a torque that would drive rotational motion by exerting a greater force on one side of an illuminated crystal. Due to the angular sensitivity of RXT, it is possible to investigate radiation pressure effects due to a non-homogeneous beam profile. It is important to decouple the temperature effects, which would change the rotational motion due to a change in the thermal energy and viscosity, as shown for glycerol above, from the pressure effects.

**Figure 2:**
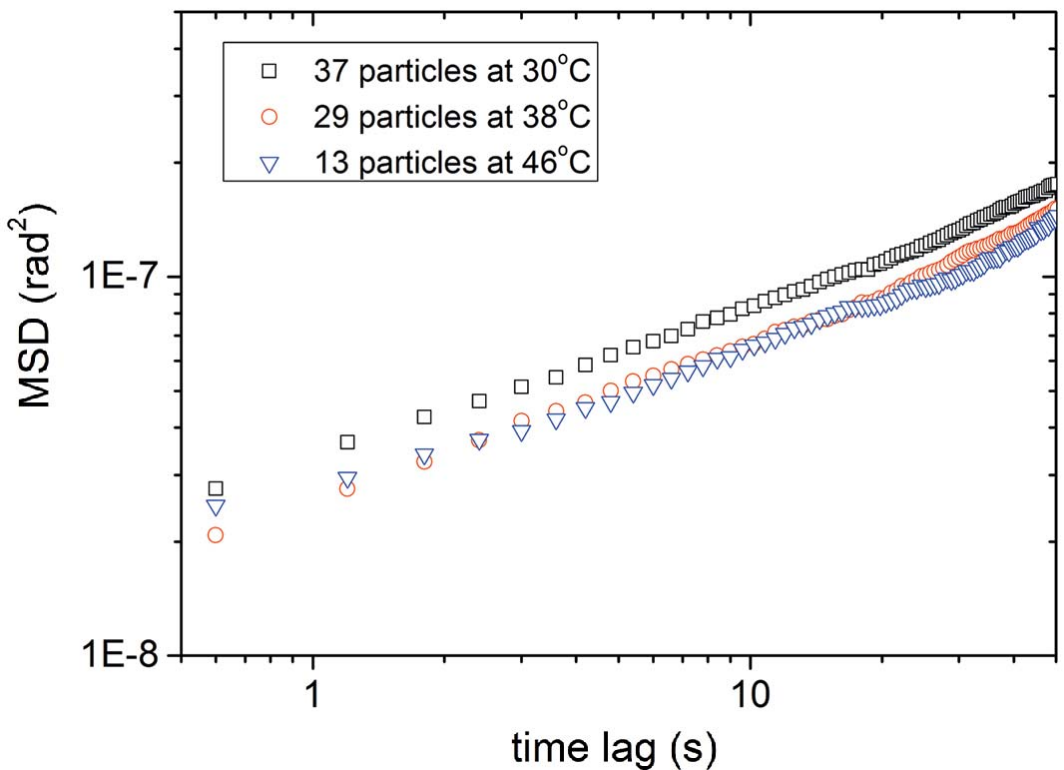
MSD *versus* time for 340 nm particles in decanoic acid at three different temperatures controlled with a Peltier heater. No systematic temperature dependence is observed.

Single-crystal 340 nm α-alumina crystals (sample detail in Liang *et al.*, 2014 ▸) were suspended in decanoic acid at a 40–50% volume fraction forming a colloidal gel (Liang *et al.*, 2014 ▸; Bell *et al.*, 2005 ▸). The colloidal gel is viscoelastic and has a viscous modulus nine orders of magnitude higher than pure decanoic acid (Liang *et al.*, 2014 ▸). The sample was measured with a five-circle diffractometer at sector 34ID-C at the Advanced Photon Source (APS), USA. The monochromatic X-ray beam was focused with a Kirkpatrick–Baez mirror pair to an approximately Gaussian profile with FWHM of ∼1.0 µm × 1.6 µm. Both 8.9 keV and 11 keV beams, with 10^9^ photons s^−1^, were incident on the droplet of suspended particles in transmission geometry. Photon flux was controlled by tapering the undulator and with attenuators. Diffraction of the (104) Bragg reflection of the alumina was measured with a Princeton Instruments charge coupled device (CCD) camera. We also utilized a temperature-controlled stage and performed experiments at constant X-ray flux for several temperatures. The MSD *versus* time results (Fig. 2 ▸) showed no systematic dependence on temperature. Thus, we conclude that temperature is not the main cause for the observed difference in MSD for the attenuated and un-attenuated X-ray beam measurements. Even with a temperature-controlled stage, there can be local heating, so the MSD studies are more conclusive to eliminate temperature dependence as the main effect on the rotational motion.

The lack of temperature dependence of the viscoelasticity in this study is consistent with studies finding that heating of silica gels irreversibly increases the elastic modulus due to restructuring to a more tightly packed structure (Wu *et al.*, 2012 ▸). These systems are distinct from the better-known thermoreversible nanoparticle gels such as silica colloidal gels where temperature dependence is accurately modelled by Naïve Mode Coupling Theory (NMCT) model (Ramakrishnan & Zukoski, 2006 ▸). Our sample preparation included heating to 50°C for >4 h to ensure proper dispersion of the alumina, which as a powder is highly aggregated, sufficient for the restructuring that would lead to our observed lack of temperature dependence.

**Figure 3:**
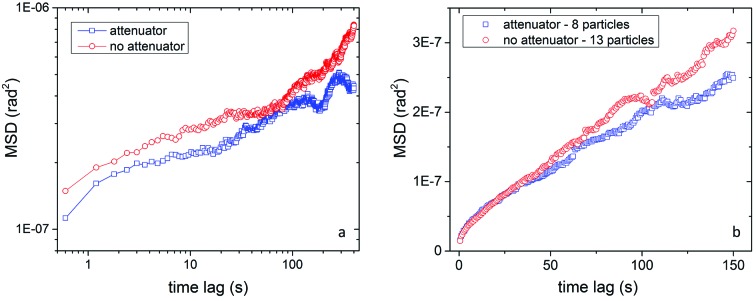
(*a*) MSD *versus* time lag for a single 340 nm alumina particle in decanoic acid with and without attenuation. (*b*) MSD *versus* time lag for an average of attenuated and non-attenuated particles.

Given that temperature appears to have little or no effect on the viscosity of the alumina/decanoic acid system, we turn our attention to effects of radiation pressure. The MSD of a single particle was studied with an X-ray beam energy of 8.9 keV at two different attenuations (Fig.3*a* ▸). A 200 µm Al attenuator (∼15% transmission) was inserted into the beam to control X-ray flux while keeping the particle in diffraction. Averaged data taken from multiple particles were also obtained to obtain better statistics. The average MSD shown in Fig. 3(*b*) ▸ was computed from data sets measured at an X-ray energy of 11 keV both with and without a 25 µm Mo attenuator (∼20% transmission). Unlike Fig. 3(*a*) ▸, these are not guaranteed to be the same particles with and without attenuator but the measurements are taken from a single sample.

In both the single-particle study and average measurements (Fig. 3 ▸) it is clear that at higher X-ray flux the rotational motions of the particles have a larger MSD *versus* time. We observe that a particle subject to a higher X-ray flux, resulting in greater radiation pressure, has a larger rotational drift component, seen as a quadratic component in the MSD.

**Figure 4:**
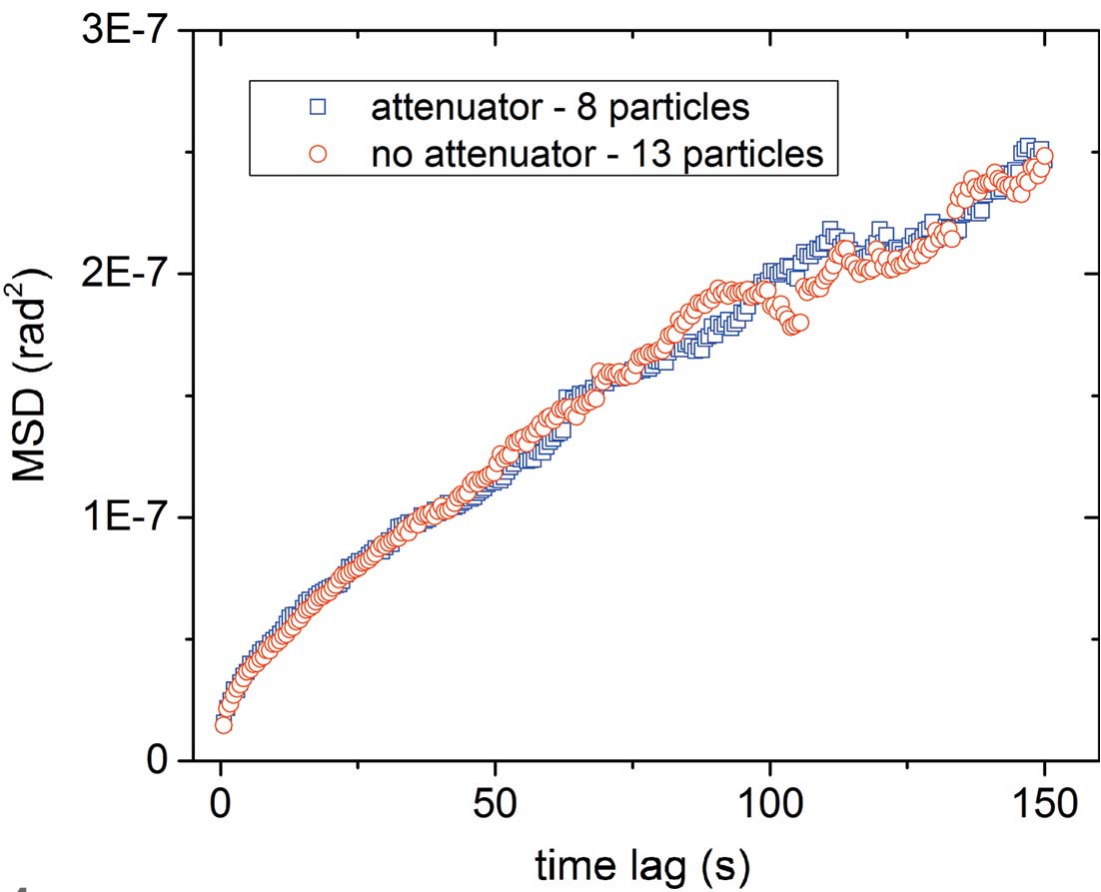
MSD *versus* time lag with the drift component subtracted for both high- and low-flux systems

Since heating was not the main contributor, the difference is due to radiation pressure. From Hasnain & Donald (2006 ▸), we treat Brownian and drift components as separate and linear. Thus we describe the MSD of the particle motion by
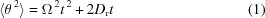
where 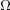 is the angular velocity and 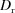 is the rotational diffusion coefficient. This angular velocity is the rotational drift of a particle due to radiation pressure, which can be determined by measuring the MSD *versus* time at the two X-ray flux levels. From Perrin (1928 ▸), we know that the rotational drift in a viscous medium is directly proportional to the torque τ. The torque of the particle with the attenuated beam, τ_2_, is related to the torque on the particle at full flux, τ_1_, by 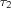 = 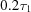. Assuming that the Brownian components are equal, the rotational drift of the low flux particle is
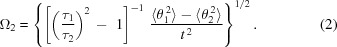
Using the MSD measured with the attenuated and non-attenuated X-ray beams, and taking the median value of 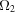, we obtain a rotational drift of 1.75 × 10^−6^ rad s^−1^ for the particle in the high-flux case and 3.5 × 10^−7^ rad s^−1^ for the low-flux case. By subtracting the drift value for both high- and low-flux MSD curves, we can obtain an estimate of the pure diffusive component, *i.e.* the MSD that would be measured without beam effects. As can be seen in Fig. 4 ▸, the two trajectories collapse on each other with the drift component removed. This represents a refinement of the RXT technique whereby we can normalize for radiation pressure effects that contribute to rotational drift.

## 4. Torque field in a Gaussian beam   

**Figure 5:**
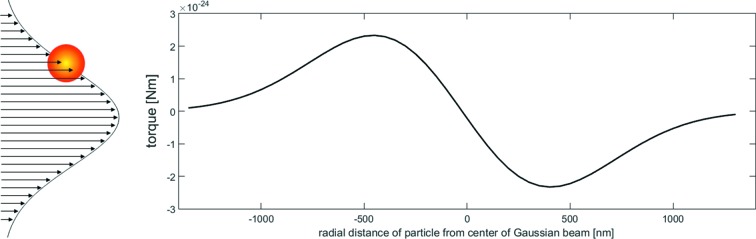
Torque on a spherical particle as a function of radial distance (nm) from the center of a Gaussian beam along a central axis of the beam.

To illustrate how radiation pressure can cause a torque, consider the torque from a surface perpendicular to a linear gradient. In reflection, the momentum transfer is in the direction of the incident beam and more incident photons on one side of a particle would induce a net torque. In our situation, with Bragg reflection of a particle in a Gaussian gradient (Fig. 5 ▸), the momentum transfer is elastic and in the direction of the scattering vector **k**_f_–**k**_i_, where **k**_i_ is the incident scattering vector and **k**_f_ is the outgoing scattering vector. The same argument for more photons on one side of the particle remains, but the direction of the net torque is no longer normal to the plane defined by the gradient direction and the incident beam direction, but rather the plane defined by the gradient direction and the scattering vector. Fig. 5 ▸ shows the magnitude of torques on a 340 nm particle at various positions along a Gaussian beam central axis, showing a maximum torque of 2 × 10^−24^ N m as computed later in this section.

An incident beam of electromagnetic radiation exerts a force on a particle both through absorption and reflection of the beam. In our case, the absorption will be minimal as the crystals are a small fraction of the extinction length of the X-rays at the wavelengths used. For a crystal oriented at the Bragg condition, the Bragg reflected beam can be quite strong. We have estimated that the (104) Bragg reflection of a 340 nm-diameter alumina particle will contain 0.15% of the incident intensity using the reflectivity of 1300 layers of the (104) Bragg planes. This value agrees within an order of magnitude with the observed intensity of the Bragg peak on the detector taking into consideration transmission through air, droplet and attenuators. Other sources of scattering, such as diffuse scattering and small-angle scattering about the direct beam, will exert a much smaller net force and be relatively isotropic compared with the highly directional nature of the Bragg beam. We do not expect contributions from the excitation of other Bragg peaks due to the α-Al_2_O_3_ crystal size and lattice parameters. An analysis showed that less than 2% of crystals oriented at one of the (104) reflections would have any contribution from another Bragg peak. The percentage is further reduced when considering that we only observe crystals that are well centered on the rocking curve resulting in strong diffraction.

**Figure 6:**
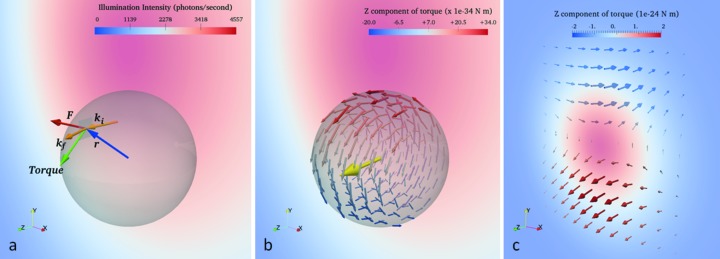
A spherical crystal in a Gaussian X-ray beam oriented at a Bragg angle will experience a radiation-pressure-induced force due to the reflected beam. Both the force and torque exerted on each volume element as a result of the Bragg scattered beam are computed as shown in (*a*). The torque vector field for each volume element of the crystal is computed (*b*). The sum gives the net torque of magnitude 2 × 10^−24^ N m, illustrated as a yellow arrow, on the crystal at a given location in the Gaussian beam (*b*). The net torque due to Bragg diffraction from a 340 nm alumina crystal at each position in a Gaussian X-ray beam (*c*). The beam is 1 µm FWHM and the Bragg angle is in the *XZ* plane. The vector color represents the *Z* component of torque on the crystal at that location (*c*).

If the particle is off center in the beam profile and undergoing Bragg diffraction to a specific direction, an asymmetric intensity gradient will exist across the particle that gives rise to a gradient in applied force, as illustrated in Fig. 6(*a*) ▸, where the sphere is sitting at the lower waist of the incident Gaussian profile beam. The top of the sphere will experience a greater net force than the bottom due to cumulative momentum transfer of the greater number of Bragg reflected photons. As a result, a net torque will be applied about the center of mass of the particle.

We estimate the torque on a 340 nm spherical particle oriented at the Bragg angle within a Gaussian beam approximating the parameters of the beam used in our RXT experiment (FWHM = 1.0 µm containing 5 × 10^9^ photons s^−1^). Fig. 6 ▸ illustrates the model used where the Bragg reflection from the crystal lies in the *x*–*z* plane, similar to the experimental geometry. The incident Gaussian beam (with direction shown as **k**_i_ in Fig. 6*a* ▸) propagates along the *z*-axis and the Bragg reflected beam is in the direction given by **k**_f_. The force on a scattering element, which we define as a 1 nm^3^ volume element of the crystal, oriented at the Bragg angle (θ_Bragg_) is given by
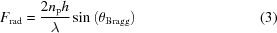
where *n*_p_ is the number of photons per second, *h* is Planck’s constant and λ is the wavelength. The force is applied in a direction perpendicular to the lattice plane and anti-parallel to the momentum transfer from the crystal to the photons. We scale the reflected intensity of each scattering element by the computed reflectivity of the (104) Bragg reflection.

The force on a volume element, shown in Fig. 6(*a*) ▸ as a red arrow, will be anti-parallel to the momentum transfer given by **k**_f_ − **k**_i_. The torque about the center of mass of the crystal (**r** × **F**) is illustrated as the green arrow in Fig. 6(*a*) ▸. Fig. 6(*b*) ▸ illustrates the torque vector field on each volume element of a particle at the lower waist of the incident beam profile. The color of the vectors in Fig. 6(*b*) ▸ describes the *z*-component of the torque exerted at that location. The net torque on the particle at this location, shown as a yellow arrow in Fig. 6(*b*) ▸, is obtained by integrating the torque vector field throughout the volume of the sphere. This approximates equation (4) which gives the net torque as a function of position *r*(*x*,*y*) in a two-dimensional Gaussian beam centered at the origin on a spherical particle of radius *R*,
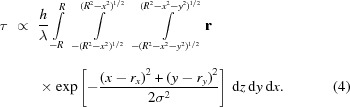
The torque is primarily along the *z*-axis at the particle location in Fig. 6(*b*) ▸ and will have the net effect of rotating the crystal on an axis almost parallel to the incident beam.

The same simulation procedure is repeated at each position in the incident Gaussian beam giving the torque on a crystal at each location, shown in Fig. 6(*c*) ▸. When the crystal is shifted along the X-axis from the center of the beam, the torque is parallel to *Y*, which rotates the crystal about the *y*-axis. This rotation will move the particle across the Debye–Scherer cone and out of diffraction of the monochromatic beam. As the crystal is shifted along the *Y*-axis, from the center of the beam, there will be a significant component of torque (on the scale of 10^−24^ N m) parallel to the X-ray beam (*z*-axis) direction. The induced rotation would cause the Bragg peak to move around the Debye–Scherrer cone, resulting in the trajectories that we measure with RXT.

## 5. Steady-state viscosity   

By modelling the torque field on the particle in a beam, we have the opportunity to glean additional information about the decanoic acid/alumina colloidal gel beyond what was obtained by Liang *et al.* (2014 ▸) by estimating the steady-state viscosity. The rotational drift, 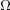, of a spherical particle of radius *R*, embedded in a medium with steady state viscosity η, subject to a torque τ, is described by 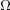 = τ/8πη*R* ^3^ (Perrin, 1928 ▸).

A colloidal gel is viscoelastic and has no simple, single viscosity value but rather viscous and elastic components that are frequency (ω) dependent. The viscoelasticity as a function of frequency, studied with traditional rheology and microrheology *via* the motion of embedded particles, is well established (Ferry, 1980 ▸; Mason & Weitz, 1995 ▸). The viscous and elastic moduli for alumina/decanoic acid gels was calculated from the MSD *versus* time previously using RXT showing good agreement with rheometry measurements (Liang *et al.*, 2014 ▸). In principle, the ω → 0 limit of the viscous moduli is an estimate for the steady-state viscosity value (Ferry, 1980 ▸) but in practice the elastic and viscous modulus of the material are often described by a power-law fit which makes the steady-state value elusive for traditional rheology data. A type of steady-state viscosity can be obtained from an estimate of the torque from the radiation pressure of a Gaussian beam as calculated above. If we assume that the particles are uniformly distributed with a cut-off at twice the FWHM (beyond which there would be little diffraction intensity from these crystals), we see that the ‘average’ torque is 1.5 × 10^−24^ N m. This torque and the full flux drift value of 1.75 × 10^−6^ rad s^−1^ found above would give a steady-state viscosity value of 7.0 Pa s for the alumina/decanoic acid colloidal gel.

In conclusion, we studied the thermal and pressure effects of synchrotron beams in systems of suspended particles by imaging the rotational motion using rotational X-ray tracking. X-ray generated thermal effects on the rotational motion of a particle are well understood given the change in viscosity and differences in thermal energy in the system. We have investigated in detail the torque caused by a Gaussian beam profile on constituent alumina particles in a colloidal gel without strong temperature-dependent viscoelasticity. The results show the effects of radiation pressure manifesting as rotational drift due to torques on the order of 10^−24^ N m. Quantifying these radiation effects also presents a way to normalize for radiation pressure from a non-uniform beam in RXT data by measuring the system at two different known fluxes.

We note that the situation of very high X-ray intensities in small focal spots will be important and more common in the future. Obtaining such ‘nanoprobe’ beams is precisely the long-term goal of many X-ray facilities, motivating machine upgrades of their electron storage rings. The driven rotation mechanism explained in this work will be exacerbated considerably with these technological advances. It represents a new damage mechanism of crystalline materials exposed to the high-flux gradients associated with strongly focused beams. It has been observed, for example, that particle rotations can be driven in materials normally considered as ‘solids’, such as baked enamel paints, where the crystalline pigment particles are found to rotate whenever they are examined with focused X-ray beams.
